# Gastrointestinal tract morphology, nutrient digestibility, and digesta mean retention time in laying hens of two different breeds fed with fine or coarse oat hulls

**DOI:** 10.1016/j.psj.2024.104333

**Published:** 2024-09-16

**Authors:** Mochammad F. Habibi, René P. Kwakkel, Sonja de Vries

**Affiliations:** ⁎ Animal Nutrition Group, Department of Animal Sciences, Wageningen University & Research, 6700 AH Wageningen, The Netherlands; † Laboratory of Poultry Science, Department of Animal Production, Faculty of Animal Science, Universitas Gadjah Mada, Sleman, Yogyakarta 55281, Indonesia

**Keywords:** chicken, insoluble fiber, passage rate, particle size

## Abstract

The inclusion of fiber-rich ingredients in poultry diets is expected to increase due to societal-, ecologic-, and economic developments. Particle size of dietary fiber sources, such as oat hulls (**OH**), has been shown to play a key-role in nutrient digestibility and digestion process, but the response may depend on the genetic background of the bird. This study aimed to test the hypothesis that laying hens varying in genetic background respond differently to the particle size of OH regarding gastrointestinal tract development, apparent ileal (**AID**) and total tract digestibility (**ATTD**), and digesta mean retention time (**MRT**). A total of 224, 29-wk-old Dekalb White (**DW**) and Bovans Black (**BB**) laying hens were assigned to 1 of 2 dietary treatments (fine vs. coarse OH). The diets were formulated to contain 150 g/kg OH as the main dietary fiber source. Titanium dioxide (3 g/kg) and cobalt-EDTA (2 g/kg) were added as inert tracers. Bovans Black hens had greater relative weights of the gizzard (+3.9% of BW, *P* = 0.015), ceca (+14.3% of BW, *P* < 0.001), and colon (+9.1% of BW, *P* < 0.001) compared with DW hens. Moreover, AID of nitrogen (+3.5%; *P* < 0.001) was greater in BB vs. DW hens, whereas AID of starch was only greater in BB when fed the fine OH diet (breed × diet, *P* = 0.037). In contrast, ATTD of NSP (-11.2%, *P* = 0.035) was lower in BB vs. DW hens and digesta MRT was longer in proventriculus + gizzard (solids: +25 min, *P* < 0.001; liquids: +5 min, *P* < 0.001) and ileum (solids: +53 min, *P* = 0.001; liquids: +38 min, *P* < 0.001). Birds fed coarse OH only had a greater relative weight of the gizzard (+3.9%, *P* < 0.001) compared with those fed fine OH. In conclusion, our study indicates that gastrointestinal tract traits, nutrient digestibility and digesta transit behavior differed between breeds, regardless of the particle size of OH in the diet.

## INTRODUCTION

For decades, laying hens have been selected for productivity traits, resulting in modern breeds with high egg production (Kidd and Anderson, 2019). At the same time, diets have been adjusted to meet the production requirements of these modern breeds, which typically contain highly digestible ingredients and low amounts of fibers (Barros de Carvalho et al., 2012; Desbruslais et al., 2021). However, due to societal-, ecologic-, and economic constraints, the poultry industry is urged to increasingly include agricultural by-products that cannot be used for human consumption, in poultry diets. These products characteristically contain high levels of dietary fibers (**DF**), which can affect digestion processes and digestibility of other nutrients (Hetland and Svihus, 2001; Jiménez-Moreno et al., 2013; Jha and Mishra 2021). For instance, various soluble DF sources have been found to influence digesta viscosity, digesta passage rate, and microbial activity in the upper gastrointestinal tract (Owusu-Asiedu et al., 2006; Bedford and Cowieson, 2012; Konieczka and Smulikowska, 2018), thereby often reducing digestion of protein and starch (Dorado-Montenegro et al., 2024). However, insoluble DF sources may improve nutrient digestibility (Gonzalez-Alvarado et al., 2007), generally ascribed to the physical structure (Naeem et al., 2023). In this respect, oat hulls (**OH**), rich in insoluble DF, have been extensively studied and shown to improve grinding activity of the muscular gizzard and stimulating enzyme secretion, resulting in improved digestion of starch (Svihus et al., 2002; Hetland et al., 2003). These properties make insoluble DF-rich by-products an attractive and competitive ingredient to include in poultry diets, despite the resulting dietary energy dilution. To optimally use DF in poultry diets and exploit their physical structure, particle size is an important factor to consider. Coarsely ground OH, were found to increase gizzard weight by 46 to 92% (Hetland and Svihus, 2001; Svihus, 2011; Sacranie et al., 2012), thereby enhancing the gizzard's grinding capacity due to increased muscular contractions and a prolonged retention time (Hetland and Svihus, 2001; Svihus, 2011; Sacranie et al., 2012; Singh et al., 2014). Eventually, a longer MRT of digesta results in longer exposure to digestive juices, and this may improve nutrient absorption (Ngoc et al., 2013; Kheravii et al., 2018).

In response to the inclusion of DF-rich sources in a diet, previous research suggested that chickens have the ability to cope with DF, but that this may vary genetically. Broiler chickens, selected for high nutrient digestion, had heavier GIT segments (proventriculus, gizzard, and ileum), longer digesta mean retention time (**MRT**) in the gizzard, and higher protein digestibility compared with chickens selected for low nutrient digestion (Rougière and Carré, 2010). Moreover, other authors found that broiler chickens representative for the broiler population in 1957, did not differ in BW and FCR when offered a diet reflecting formulation practices from 1957 with the inclusion of DF (alfalfa meal and wheat middlings), compared with a modern (high-energy and low-fiber) diet, whereas modern-type broilers grew less fast and had impaired FCR when such a diet was fed (Havenstein et al., 2003). Similarly, fast-growing broilers (Cobb500) had higher CP digestibility and metabolizability of acid detergent fiber compared with slow-growing broilers (Label Rouge) only when they were fed with a low-fiber (3 g/kg) diet, but not when fed a high-fiber (5 g/kg) diet (Krás et al., 2013).

Based on the above-mentioned findings, it may be postulated that the response of chickens to fiber particle sizes on the development of the digestive tract, nutrient digestibility, and digesta retention time, may vary among breeds. Although previous studies clearly indicated that (artificially selected) broiler breeds varying in production or digestion efficiency, differ in their ability to respond to high-fiber diets, little is known about the variation in responses among modern commercial breeds, particularly in laying hens. Therefore, this experiment aimed to investigate whether 2 commercial laying hen breeds, Dekalb White and Bovans Black, which selected for different production traits, respond differently when fed with fine or coarse OH, as an insoluble DF source, regarding digestive tract development, nutrient digestibility, and digesta MRT. It was hypothesized that coarse OH will increase GIT weight, especially of the gizzard, improve nutrient digestibility, and prolong digesta MRT in proventriculus + gizzard compared with fine OH, but that the responses may differ between the 2 breeds.

## MATERIALS AND METHODS

### Birds, Housing, and Diets

This study was conducted at the research facilities of Wageningen University & Research, and all experimental procedures were approved by the Animal Welfare Body of Wageningen University & Research and the Dutch Central Committee of Animal Experiments, The Netherlands, under the authorization number AVD1040020197324.

The effect of coarseness of OH (fine vs. coarse) was tested in 2 laying hen breeds (Dekalb White vs. Bovans Black; Hendrix Genetics, Boxmeer, The Netherlands) using a 2 × 2 factorial arrangement of treatments. A total of 112, 29-wk-old Dekalb White (**DW**) (BW: 1.53 ± 0.107 kg) and 112, 29-wk-old Bovans Black (**BB**) (BW: 1.79 ± 0.127 kg) laying hens were housed in 32 pens (1.85 × 1.10 m, 7 birds/pen) with floating plastic slatted floors, covered with rubber matting and wood shavings. Pens were located in 3 rooms, with 10 or 12 pens per room. Breed- and diet treatment combinations were balanced over rooms. Within the room, pens were assigned alternately to 1 of the 2 breeds. Within breed, pens were randomly assigned to 1 of 2 experimental diets. Diets were formulated to contain 150 g/kg fine (ground using a hammermill (LHM20/16, 1.5 kW, Condux International, Mankato, United States of America) at 1500 rpm using a 1.5 mm screen) or coarse (intact) oat hulls (**OH**) as the main DF source. Diets were fed as pellets and formulated to meet or exceed nutrient requirements for laying hens (Table 1; CVB, 2018) and fed as pellets. Titanium dioxide (TiO_2_; 3 g/kg), and cobalt-ethylenediaminetetraacetic acid (Co-EDTA; 2 g/kg) were included in the diet as inert tracers for fine-solid (hereafter referred to as solid) and liquid digesta, respectively. Birds had *ad libitum* access to feed and water. Room temperature was controlled at 20°C and relative humidity at 60 to 70%. The photoperiod was 15L:9D and the maximum light intensity was 10 lux at bird level. At d 24 of the experiment, bedding and rubber matting were removed and birds were housed on slatted floors.

**Table 1:** Ingredient and nutrient composition of experimental diets containing oat hulls varied in particle size^1^ (g/kg, as-fed basis).

Item	Diet
Ingredient	
Maize	525.5
Oat hulls^1^	150.0
Wheat gluten	100.0
Toasted soybeans	50.0
Soy oil	42.0
Mineral and vitamin premix^2^	5.0
CaCO_3_	90.0
Dicalcium phosphate	18.0
Salt	1.0
NaHCO_3_	3.6
MgO	1.0
L-Lysine HCl 79%	5.0
DL-Methionine	1.4
L-Threonine	1.3
L-Valine	0.2
L-Tryptophan	0.4
L-Isoleucine	0.6
TiO_2_	3.0
Co-EDTA	2.0
Calculated nutrient composition^3^	
AME^4^ (kcal/kg)	2916
Digestible lysine^5^	7.6
Digestible methionine + cysteine^5^	6.9
Digestible threonine^5^	5.3
Digestible tryptophan^5^	1.6
Calcium	39.1
Phosphorus	3.0
Analyzed nutrient composition	
Dry matter (g/kg)^6^	902.2
CP^7^ (g/kg in DM)	169.6
Fat (g/kg in DM)	53.8
Starch (g/kg in DM)	390.8
Total Nonstarch polysaccharides (g/kg in DM)	128.5
Of which soluble (g/kg in DM)	14.3
Of which insoluble (g/kg in DM)	114.2

1 Oat hulls were either used ground fine by hammer milling using a 1.5mm sieve (fine) or intact (coarse).

2 Provided per kilogram of diet: Vitamin A (retinyl acetate), 10,000 IU; vitamin D (cholecalciferol), 2,000 IU; vitamin E (dl-a-tocopherol), 25 mg; vitamin K_3_ (menadione), 1.5 mg; vitamin B_1_ (thiamin), 1 mg; vitamin B_2_ (riboflavin), 3.5 mg; vitamin B_6_ (pyridoxine-HCl), 15 μg; niacin, 30 mg; D-pantothenic acid, 12 mg; choline chloride, 350 mg; folic acid, 0.8 mg; biotin, 0.1 mg; iron, 50 mg; copper, 10 mg; manganese, 60 mg; zinc, 54 mg; iodine, 0.7 mg; selenium, 0.1 mg.

3 Calculated using data from (CVB, 2018).

4 Apparent metabolizable energy for laying hens (12.2 MJ/kg).

5 Standardized ileal digestible amino acids for laying hens.

6 The DM of the fine OH diets was 908.2 g/kg.

7 Nitrogen × 6.25.

### Sample Collection

Excreta were quantitatively collected at d 28, 29, 30, and 31 per pen. Subsamples of clean excreta were taken and frozen immediately in the freezer at −20°C. At d 34, 35, or 36, birds were euthanized, weighed, and dissected according to a dissection schedule balancing treatments and timepoints for cecal digesta transit measurements over days and time of the day. Individual BW of birds was recorded prior to dissection. Subsequently, each individual bird received an injection of 0.5 mL sodium pentobarbital (20% or 500 mg/mL) administered at the base of the posterior edge of the skull. The abdominal cavity was opened and, if present, fully developed eggs in the distal oviduct were removed and weighed to correct BW. Then the GIT from esophagus to rectum, was removed and crop, gizzard + proventriculus, duodenum + jejunum, ileum, and ceca + colon were dissected and weighed. For each segment, digestive contents were collected on ice, by gentle-finger stripping, and frozen at −20°C. Empty GIT segments were flushed with water, dried with paper tissue, and weighed. The length of small intestine, ceca, and colon was measured using measuring tape. Before freeze-drying, excreta and digesta samples were thawed (4°C) and pooled per pen.

### Cecal Digesta Transit

To assess cecal digesta transit, a pulse dose of Cr-mordanted OH (1 g) and ytterbium acetate (250 mg) markers was provided to individual birds between d 34 to 36. Coarse and fine OH were mordanted with Cr_2_O_3_ according to the procedure described by Dorado-Montenegro et al. (2023). Ytterbium acetate is soluble in water, but tends to associate with particulate matter (Siddons et al., 1985). The markers were incorporated into a small amount of feed mixed with water and rolled into spheric boluses of ∼1.5 cm diameter (∼ 5 g/pulse dose, divided into 4–6 boluses). Each set of 2 pens of the same treatment within a block of 8 pens, was considered 1 experimental unit (4 replicates/treatment). The fourteen birds of 1 experimental unit were randomly assigned to 1 of the 14 timepoints. At t = 0 (d 34, d 35, or 36), the marker pulse dose boluses were gently dosed into the oropharyngeal cavity by hand. Birds were subsequently euthanized at after 30, 60, 120, 180, 240, 300, 360, 480, 600, 720, 960, 1,200, 1,440, and 1,800 min after the pulse dose.

### Analytical Methods

#### Wet Sieve Analysis

**Figure 1:**
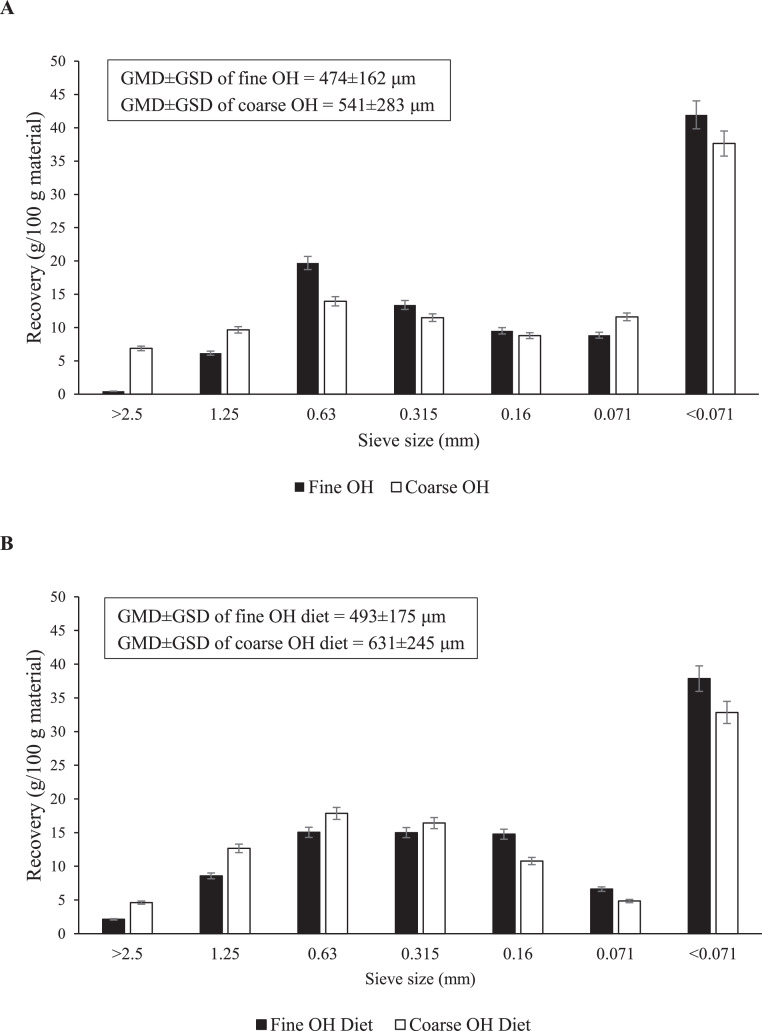
Weight fraction of fine and coarse oat hulls (OH) (A) and OH diets (B) recovered at sieves with various mash sizes during wet sieve analyses. Bars represent mean of 2 replicate measurements. Error bars represent SEM. GMD: geometric mean diameter, GSD: geometric standard deviation.

Particle size distribution of OH and OH diets was analyzed by wet sieving in duplicate (Wolf et al., 2010) using sieve sizes 2.5, 1.25, 0.63, 0.315, 0.160, and 0.071 mm. The geometric mean diameter (**GMD**) and geometric standard deviation (**GSD**) of the OH diets were calculated as described by Lyu et al. (2020). The GMD±GSD of fine OH was 474±162 μm and of coarse OH was 541±283 μm . This resulted in a GMD±GSD of 493±175 μm for the fine OH diet and 631±245 μm for the coarse OH diet (Figure 1).

#### Chemical Analyses

Prior to chemical analyses, digesta and excreta samples were freeze-dried and moisture loss was recorded. Feed-, digesta-, and excreta samples were ground by a rotor mill (Retsch ZM200, Haan, Germany) at 12,000 rpm using a 1 mm screen. All analyses were performed in duplicate. Samples were analyzed for contents of DM (AOAC, 2016; method 930.15 for feed and excreta; due to limited sample quantity, DM of digesta was derived from the moisture loss during freeze-drying), nitrogen (ISO 16634, 2008), ether extract (after hydrochloric acid hydrolysis, using Soxhlet apparatus and petroleum ether; AOAC, 2016; method 920.39), and total starch (ISO 15914, 2004). Total nonstarch polysaccharides (**NSP**) were measured as neutral sugars and uronic acids, according to the method of Englyst et al. (1994), with some modifications as described by Lannuzel et al. (2024). For feed samples, starch was gelatinized and enzymatically degraded prior to NSP extraction. Inositol and allose were used as internal standards. Uronic acid concentration was analyzed according to the colorimetric m-hydroxydiphenyl assay (Blumenkrantz and Asboe-Hansen, 1973), using a spectrophotometer (Evolution 201, Thermo Scientific, Waltham, MA). Galacturonic acid was used for calibration. Titanium (**Ti**), cobalt (**Co**), chromium (**Cr**), and ytterbium (**Yb**) were analyzed after ashing and microwave digestion using an Avio 500 inductively coupled plasma optical emission spectrometer (Perkin Elmer, Waltham), as described by Lannuzel et al. (2024).

### Calculation of Digestibility and Mean Retention Time

Apparent ileal digestibility (**AID**) and apparent total tract digestibility (**ATTD**) of DM, nitrogen, fat, starch, and NSP were calculated, as follows (de Vries and Gerrits, 2018):

$$AID_{X}\;or\;ATTD_{X}\left(\%\right)=\left(1-\frac{\left[T\right]diet\;\times \;\left[X\right]sample}{\left[T\right]sample\;\times \;\left[X\right]diet}\right)\times 100\%$$

where [T]diet, [T]sample, [X]diet, and [X]sample (g/kg) are concentrations of the tracer (T) (titanium) and nutrient (X) in the diet, digesta, or excreta samples. Digesta MRT was calculated from ingestion of tracer T during 24 h (intake/1440 min, g) and the pool size of tracer T detected in the digesta sample at dissection, assuming steady state conditions under *ad libitum* feeding strategies (van der Klis et al., 1990):

$$MRT\;\left(min\right)=1440\times \left(\frac{\left[T\right]sample\times \;Wsample}{\left[T\right]diet\;\times \;intake/1,440\;min}\right)$$

where [T] is concentrations of the tracer Ti (g/kg) and Co (mg/kg) in the diet and digesta sample, and Wsample (g) is the digesta pool size. The marker intake was calculated from the average of feed intake from d 11 to 31 for each bird.

### Time of Peak of Marker Concentration in the Ceca

Time of peak of Cr and Yb concentrations in the ceca after providing a pulse dose, was estimated by fitting (PROC NLIN, SAS, version 9.4, SAS Institute Inc., Cary, NC) a generalized Michaelis-Menten equation based on van Den Borne et al. (2007):

$$y=\;\frac{\left[a\;\times c\;\times t^{\left(-c-1\right)}\times b^{c}\right]}{{\left[1+{\left(\frac{b}{t}\right)}^{c}\right]}^{2}}$$

where y = the marker concentration (chromium or ytterbium) in cecal digesta (mg/kg) at time t (min); a, b, and c are parameters that define the curve. Then, the time of peak (min) was calculated as t_max_ = [c^b^·(1-b)/(-b-1)]^(1/b)^. The concentration at time of peak (maximum marker concentration, mg/kg) was then calculated as y_max_ = a·b·t_max_^(-b-1)^ ·c^b^/[1+(c/t_max_)^b^]^2^.

### Statistical Analysis

The pen was the experimental unit for statistical analyses of GIT traits, nutrient digestibility, digesta MRT, and time of peak. These data were analyzed by 2-way ANOVA (PROC GLM, SAS, version 9.4, SAS Institute Inc., Cary, NC), with breed (BB vs. DW), diet (fine vs. coarse), and their interaction as fixed effects. In case of significant breed × diet interactions, differences among means were assessed using Tukey adjustments. The set of 2 pens containing 14 birds dissected at 14 consecutive timepoints after marker pulse dose was considered the experimental unit for statistical analysis of cecal digesta transit. Effects of breed, diet, and their interaction on the estimated time of peak of Cr and Yb concentrations were analyzed using the model described above. Subsequently, differences in the estimated time of peak between markers (Cr vs. Yb) were evaluated. Preliminary analyses revealed no interactions between breed, diet, and marker and hence data were pooled over breeds and diets. Differences in estimated time of peak and estimated concentration at time of peak between markers were tested using a paired t-test (PROC GLM, SAS, version 9.4, SAS Institute Inc., Cary, NC) with marker (chromium vs. ytterbium) as a fixed effect. Relations among AID of nitrogen and MRT in proventriculus + gizzard and small intestine were evaluated with Pearson correlation coefficients (PROC CORR, SAS, version 9.4, SAS Institute Inc., Cary, NC), where pen was considered as experimental unit. For all statistical analyses, homogeneity and normality of model assumptions were visually verified using histograms, QQ-plots, and studentized residuals. Data are presented as means and pooled SEM. Differences were considered significant at *P* < 0.05.

## RESULTS

### Body Weight, Feed Intake, and Gastrointestinal Tract Traits

Body weight of BB hens was greater than that of DW hens (1,624 vs. 1,541 g, *P* < 0.001), whereas relative daily feed intake was smaller (41 vs. 47 g/d per kg BW, *P* < 0.001) (Table 2). Diet did not influence BW nor feed intake. BB hens had smaller liver (-2.1 g/kg BW, *P* < 0.001), pancreas (-0.4 g/kg BW, *P* < 0.001), crop (-0.3 g/kg BW, *P* = 0.007), and proventriculus (-0.1 g/kg BW, *P* = 0.046) than DW hens. Empty relative weights of gizzard (+1.4 g/kg BW, *P* = 0.015), ceca (+0.7 g/kg BW, *P* < 0.001), and colon (+0.2 g/kg BW, *P* = 0.019) were greater for BB than for DW hens. Moreover, BB hens had shorter ileum (37.4 vs. 40.1 cm/kg BW, *P* < 0.001) and longer ceca (10.6 vs. 9.1 cm/kg BW, *P* < 0.010) compared with DW hens. Relative digesta weights of proventriculus + gizzard (9.8 vs. 8.3 g/kg BW, *P* < 0.001), ileum (5.4 vs. 4.8 g/kg BW, *P* = 0.026), and ceca (1.1 vs. 0.8 g/kg BW, *P* = 0.005) of BB hens were larger, but the relative weight of digesta in the crop (10.9 vs. 15.5 g/kg BW, *P* < 0.001) was smaller, compared with DW hens. When fed the fine OH diet, empty relative weights of the gizzard (19.6 vs. 20.8 g/kg BW, *P* < 0.001) and digesta in ileum (4.8 vs. 5.4 g/kg BW, *P* = 0.040) were smaller compared with the coarse OH diet. In contrast, particle size had no effect on empty relative weights and lengths of other empty GIT tract segments, nor on relative weights of digesta contents.

**Table 2:** Body weight, feed intake, digestive organ size, and digesta weight in Dekalb White or Bovans Black laying hens fed diets containing fine vs. coarse oat hulls (150g/kg).^1^

Item	Dekalb White		Bovans Black		SEM	Model established *P*-values^2^		
	Fine	Coarse	Fine	Coarse		Breed	Diet	B×D
n^3^	8	8	8	8				
Body weight (g)^4^	1,547	1,534	1,647	1,600	18.5	<0.001	0.115	0.380
Relative feed intake (g/kg BW/d)	47	47	40	41	1.5	<0.001	0.705	0.899
Relative organ weight (g/kg BW)								
Liver	27.0	26.7	24.6	25.0	0.51	<0.001	0.895	0.478
Pancreas	2.3	2.3	1.9	1.8	0.05	<0.001	0.449	0.556
Crop	3.7	3.8	3.4	3.6	0.10	0.007	0.101	0.632
Proventriculus	3.8	3.8	3.6	3.7	0.06	0.046	0.259	0.616
Gizzard	19.1	20.5	20.1	21.0	0.31	0.015	<0.001	0.499
Duodenum + Jejunum	22.0	21.9	21.8	22.3	0.48	0.434	0.289	0.229
Ileum	8.6	8.5	8.0	8.4	0.27	0.211	0.436	0.324
Ceca	4.3	4.1	4.8	4.9	0.14	<0.001	0.971	0.213
Colon	2.0	2.0	2.1	2.2	0.06	0.019	0.188	0.394
Relative organ length^5^ (cm/kg BW)								
Duodenum + Jejunum	57.5	57.4	57.4	58.7	0.90	0.622	0.445	0.373
Ileum	39.6	40.6	36.5	38.2	0.75	<0.001	0.071	0.568
Ceca	8.8	8.7	10.1	9.8	0.25	<0.001	0.468	0.629
Colon	5.1	5.4	5.3	5.4	0.23	0.812	0.430	0.522
Relative weight of digesta (g/kg BW)								
Crop	14.8	14.3	8.6	11.3	1.19	<0.001	0.171	0.365
Proventriculus + Gizzard	8.3	8.3	9.6	10.0	0.30	<0.001	0.391	0.555
Duodenum + Jejunum	5.9	6.6	6.4	6.8	0.29	0.262	0.053	0.719
Ileum	4.5	5.0	5.0	5.7	0.27	0.026	0.040	0.588
Ceca	0.8	0.7	1.1	1.0	0.09	0.005	0.336	0.972
Colon	1.7	1.6	1.8	1.7	0.24	0.612	0.519	0.982

1 Oat hulls were either used ground fine by hammer milling using a 1.5mm sieve (fine) or intact (coarse).

2 Model established *P*-values for the fixed effects of breed, oat hull particle size, or their interaction (B×D).

3 Number of replicate pens (5 to 7 birds/pen, unless indicated otherwise).

4 Body weight was corrected for egg weight inside the body during the dissection day.

5 Three birds/pen.

### Apparent Ileal and Total Tract Digestibility

Bovans Black hens had higher AID of DM (62.7 vs. 60.7%, *P* = 0.033) and nitrogen (81.9 vs. 78.4%, *P* < 0.001) than DW hens (Table 3). The AID of starch was only different between breeds when the fine OH diet was fed (breed × diet, *P* = 0.037). Contrastingly, BB hens had a lower ATTD of DM (62.8 vs. 64.9%, *P* < 0.001), nitrogen (39.1 vs. 47.1%, *P* < 0.001), fat (83.2 vs. 87.4%, *P* = 0.001), and NSP (6.4 vs. -2.4%, *P* = 0.035) than BB hens. No differences in AID nor ATTD between diets were observed.

**Table 3:** Apparent ileal (AID) and total tract (ATTD) digestibility (% of intake) of dry matter, nitrogen, fat, starch, and nonstarch polysaccharides (NSP) in Dekalb White or Bovans Black laying hens fed diets containing fine vs. coarse oat hulls (150 g/kg).^1^

Item	Dekalb White		Bovans Black		SEM	Model established *P*-values^2^		
	Fine	Coarse	Fine	Coarse		Breed	Diet	B×D
n^3^	8	8	8	8				
AID								
Dry matter	59.7	61.6	62.4	62.9	0.89	0.033	0.189	0.457
Nitrogen	77.8	78.9	81.8	82.0	0.88	<0.001	0.467	0.644
Fat	86.4	85.8	85.3	84.8	1.75	0.551	0.744	0.986
Starch	96.5^b^	97.5^ab^	98.2^a^	97.3^ab^	0.44	0.101	0.940	0.037
ATTD								
Dry matter	64.1	65.7	62.9	62.6	0.65	<0.001	0.120	0.274
Nitrogen^4^	46.5	47.6	39.2	38.9	1.30	<0.001	0.735	0.600
Fat	87.3	87.5	82.1	84.3	1.14	0.001	0.284	0.392
Starch	97.4	97.9	97.7	97.9	0.25	0.426	0.172	0.524
NSP^5^	4.9	7.9	-3.8	-0.9	3.80	0.035	0.450	0.989

1 Oat hulls were either used ground fine by hammer milling using a 1.5mm sieve (fine) or intact (coarse).

2 Model established *P*-values for the fixed effects of breed, oat hulls particle size, or their interaction (B×D).

3 Number of replicate pens (7 birds/pen).

4 Apparent total tract retention of nitrogen, calculated as the difference between nitrogen intake and (urinary + fecal) nitrogen in excreta.

5 Number of replicate pens for coarse diet in both breeds = 7 and SEM = 4.06.

### Digesta Passage Behavior

In BB hens, solid digesta retained longer in proventriculus + gizzard (55 vs. 30 min, *P* < 0.001), duodenum + jejunum (52 vs. 33 min, *P* < 0.001), ileum (79 vs. 57 min, *P* = 0.001), and total GIT (311 vs. 255 min, *P* = 0.021) compared with DW hens (Table 4). Liquid digesta retained longer in proventriculus + gizzard (14 vs. 9 min, *P* < 0.001), duodenum + jejunum (26 vs. 15 min, *P* < 0.001), ileum (36 vs. 26 min, P < 0.001), ceca + colon (137 vs. 77 min, *P* < 0.001), and total GIT (256 vs. 169 min, *P* < 0.001) in BB hens compared with DW hens. No differences in MRT of solid and liquid digesta between birds fed coarse vs. fine OH were observed in any of the GIT segments. The estimated time of peak of marker concentrations (chromium and ytterbium) in the ceca of birds (Table 5) did not vary among treatments.

**Table 4:** Mean retention time (MRT) (min) of solid and liquid digesta in Dekalb White or Bovans Black laying hens fed diets containing fine vs. coarse oat hulls (150 g/kg).^1^

Item	Dekalb White		Bovans Black		SEM	Model established *P*-values^2^		
	Fine	Coarse	Fine	Coarse		Breed	Diet	B×D
n^3^	8	8	8	8				
Solid digesta (min)								
Crop	108	118	88	106	11.9	0.199	0.260	0.708
Proventriculus + Gizzard	30	31	56	56	4.3	<0.001	0.943	0.984
Duodenum + Jejunum	34	34	53	53	3.3	<0.001	0.931	0.962
Ileum	56	62	79	82	5.8	0.001	0.508	0.777
Ceca + Colon	28	24	37	29	4.0	0.105	0.161	0.773
Total	256	268	312	324	22.8	0.021	0.592	0.945
Liquid digesta (min)								
Crop	84	81	78	84	4.7	0.871	0.882	0.645
Proventriculus + Gizzard	16	18	25	27	0.9	<0.001	0.439	0.977
Duodenum + Jejunum	26	28	47	50	1.9	<0.001	0.489	0.868
Ileum	47	52	68	69	2.5	<0.001	0.609	0.658
Ceca + Colon	155	137	284	238	12.3	<0.001	0.179	0.551
Total	328	316	503	467	17.3	<0.001	0.462	0.729

1 Oat hulls were either used ground fine by hammer milling using a 1.5mm sieve (fine) or intact (coarse).

2 Model established *P*-values for the fixed effects of breed, oat hulls particle size, or their interaction (B×D).

3 Number of replicate pens (7 birds/pen).

**Table 5:** Estimated time of peak (min) of marker concentrations in the ceca of Dekalb White or Bovans Black laying hens fed diets containing fine vs. coarse oat hulls (150 g/kg).^1^

Item	Dekalb White		Bovans Black		SEM	Model established *P*-values^2^		
	Fine	Coarse	Fine	Coarse		Breed	Diet	B×D
n^3^	4	4	4	4				
Chromium	1276	1173	1605	1245	278.1	0.485	0.422	0.653
Ytterbium	1123	1185	1357	1235	124.2	0.274	0.815	0.472

1 Oat hulls were either used ground fine by hammer milling using a 1.5mm sieve (fine) or intact (coarse).

2 Model established *P*-values for the fixed effects of breed, oat hulls particle size, or their interaction (B×D).

3 Number of replicate timepoints (4 birds/timepoint).

## DISCUSSION

This experiment was conducted to examine the effects of feeding high-fiber diets containing 150 g/kg OH varying in particle size (fine and coarse) on GIT traits, nutrient digestibility, and digesta MRT in 2 laying hen breeds (Dekalb White and Bovans Black). We hypothesized that coarse OH would increase gizzard weight, improve nutrient digestibility, and prolong digesta MRT in proventriculus + gizzard compared with fine OH, but that the responses would differ between the 2 breeds. Unlike our expectations, marginal diet effects were observed, despite the notable particle size contrast of the OH. Interestingly, considerable breed-related variation in gastrointestinal traits, digesta retention time, and digestibility were observed, regardless of OH particle size.

### Digesta Passage throughout the Gastrointestinal Tracts

Although digesta transit behavior in chickens is limitedly understood, it is clear that various digesta fractions may separate as they pass through the gastrointestinal tract (de Vries et al., 2014; Garçon et al., 2023; Dorado-Montenegro et al., 2024). For instance, the grinding activity in the gizzard of chickens facilitates particle size reduction of digesta before entering the small intestine (Hetland et al., 2002) and in this process, liquid digesta is expected to flow more rapidly into the small intestine than solid digesta (Garçon et al., 2023). In the hindgut, retrograde peristaltic contractions may provoke digesta segregation and are responsible for transporting liquid digesta from the cloaca to the ceca (Björnhag, 1989; Vergara et al., 1989; Clench and Mathias, 1995; Braun, 1999; Dorado-Montenegro et al., 2024). Although the filling and emptying processes of the ceca have been scarcely studied, it has been identified that solid digesta, particularly larger particles, only limitedly enter the ceca, as illustrated by the presence of 12-fold higher concentrations of liquid- vs. solid digesta tracers typically found in this segment (Vergara et al., 1989; de Vries et al., 2014). In addition, it seems that only a fraction of the liquid digesta is transported into the ceca, with provisional estimates ranging from 30 to 50% (Vergara et al., 1989; Garçon et al., 2023). Those findings are in line with the results in our study, where Co concentrations in the ceca—originating from the soluble Co-EDTA tracer - were ∼6 times higher than Ti concentrations – originating from the solid digesta tracer TiO_2_ (data not shown).

Most studies quantifying digesta retention time in chickens considered only solid digesta by using TiO_2_ or ^103^ruthenium phenanthroline as tracers (Shires et al., 1987; Dänicke et al., 1999; Rougière and Carré, 2010; van Krimpen et al., 2011; Duve et al., 2011; Moquet et al., 2018; Dorado-Montenegro et al., 2024). Only a few studies have looked into the MRT of liquid digesta throughout the gastrointestinal tract of broilers (Garçon et al., 2023; Dorado-Montenegro et al., 2024). Additionally, it was revealed that the cecal retention times of liquid digesta that actually enter the ceca can be considerable (∼500–1,500 min; Garçon et al., 2023). To our knowledge, the current study is the first investigation into the retention time of not only solid, but also liquid digesta fractions throughout the GIT of laying hens. Overall, the MRT of solid digesta observed in our study was in the same range as previously reported (Shires et al., 1987; van Krimpen et al., 2011) and the MRT of liquid digesta throughout GIT was also consistent with the range expected, based on previous studies in broilers (Garçon et al., 2023; Dorado-Montenegro et al., 2024).

### Digestion Processes in Two Laying Hen Breeds

In the current study, considerable differences in prececal digestive processes between breeds were found. Bovans Black hens showed a higher AID of nitrogen, coinciding with a lower feed intake and longer MRT of solid and liquid digesta in the proventriculus + gizzard and small intestine, compared with Dekalb White hens. The negative correlation between feed intake and MRT of digesta in the gizzard and small intestine (Table 7), supports suggestions of previous authors that lower feed intake facilitates more and prolonged contact between digesta, enzymes, and intestinal mucosa and may lead to a higher digestibility of nitrogen (Guixin et al., 1995; González-Alvarado et al., 2008; Svihus, 2011; Ngoc et al., 2013; Svihus and Itani 2019).

Surprisingly, BB hens had a lower ATTD of NSP, despite their heavier ceca and colon, and a longer MRT of liquid digesta in those segments compared with DW hens. The ATTD of total NSP was low as expected from the dietary composition. Nevertheless, interesting differences between breeds were observed, as fermentation of NSP was virtually zero in BB hens, but reached 5 to 8% of NSP in DW hens. Although OH was the major source providing dietary NSP (∼56% of total NSP in the diet), NSP also originated from maize (∼7% of total NSP in the diet) and soybeans (∼37% of total NSP in the diet). Considering the limited cecal access of OH observed in our study—as indicated by the low concentrations of Cr found in the ceca (Table 6)—and the poor fermentability of NSP from OH, the difference in the ATTD of NSP between breeds should thus most likely be ascribed to degradation of NSP from maize and soybeans.

**Table 6:** Estimated time of peak (T_max_, min) and maximum concentration (Y_max_, mg/kg DM) between markers in the ceca of laying hens fed diets containing oat hulls (150 g/kg).

Item	Chromium	Ytterbium	SEM	Model established *P*-values^1^
n^2^	16	16		
T_max_ (min)	1325	1225	102.2	0.495
Y_max_ (mg/kg DM)	1022	4099	224.4	<0.001

1 Model established *P*-values for the fixed effects of marker.

2 Number of replicate pens.

**Table 7:** Pearson correlation coefficients of AID of Nitrogen and MRT of prececal digesta.^1^^,^^2^^,^^3^

Parameters	AID of Nitrogen	MRT of solid digesta in proventriculus + gizzard	MRT of liquid digesta in proventriculus + gizzard	MRT of solid digesta in duodenum + jejunum	MRT of liquid digesta in duodenum + jejunum	MRT of solid digesta in ileum	MRT of liquid digesta in ileum	Relative feed intake
AID of Nitrogen	1.00							
MRT of solid digesta in proventriculus + gizzard	0.36*	1.00						
MRT of liquid digesta in proventriculus + gizzard	0.52†	0.89†	1.00					
MRT of solid digesta in duodenum + jejunum	0.44†	0.84†	0.79†	1.00				
MRT of liquid digesta in duodenum + jejunum	0.52†	0.81†	0.83†	0.94†	1.00			
MRT of solid digesta in ileum	0.50†	0.75†	0.79†	0.81†	0.83†	1.00		
MRT of liquid digesta in ileum	0.51†	0.75†	0.78†	0.76†	0.81†	0.97†	1.00	
Relative feed intake	−0.24	−0.74†	−0.13	−0.18	−0.61†	−0.19	−0.62†	1.00

1 Parameters n = 32 pen observations.

2 AID: apparent ileal digestibility; MRT: mean retention time.

3 *P ≤ 0.05; †*P* ≤ 0.01.

Differences in DF fermentation speculatively may point out differences in the cecal microbiome between the 2 breeds of birds. However, it should be noted that differences in ileal nutrient digestibility and feed intake between the breeds also imply that the quantity and composition of digesta flowing into the hindgut, which were different, thereby presumably affecting amount and type of substrate available for the cecal microbiome and most likely microbial activity. The lower AID of nitrogen and numerical lower AID of fat observed for BB hens may thus indirectly have contributed to differences in NSP digestibility observed. Provisional calculations indicate that in BB hens, substrate flow into the hindgut was ∼3 to 4 g/day less compared with DW hens, almost exclusively explained by a difference in nitrogen. The selective entry of only certain digesta fractions into the ceca complicates quantitative estimates of substrate flow into the ceca. Nevertheless, considering that the ratios between markers for the various digesta fractions in cecal contents did not notably vary between the 2 breeds, it seems reasonable to assume that any differences in substrate passing the ileocecocolic junction reflect differences in cecal substrate flow. The lower inflow of nitrogen in BB hens may have steered fermentation processes, thereby affecting hindgut disappearance of NSP and other nutrients as illustrated by the observed differences in ATTD of NSP and fat between BB and DW hens.

### Effects of Oat Hull Particle Size on Digestion Processes

Grinding of OH, as a model for insoluble DF, considerably reduced the fraction of coarse particles; in the coarse OH and OH diet, ∼17% (w/w) of the particles was found to be ≥ 1.25 mm, whereas this fraction was reduced to ∼7% in fine OH and ∼10% in the final fine OH diet (Figure 1). Birds fed the coarse OH diet had ∼5.8% heavier gizzard weight than those fed the fine OH diet, as also reported by van Krimpen et al. (2011) and Naeem et al. (2023). This increased gizzard weight is assumed to be the result of the accumulation of coarse OH that provokes the grinding activity (Sacranie et al., 2012), thereby resulting in an increased size of the 2 pairs of gizzard muscles (Jha and Mishra, 2021; Svihus, 2011). It could be expected that an increased gizzard weight in response to coarse OH would also result in a prolonged MRT of digesta, especially of coarse particles, in that segment. However, we did not observe differences in MRT in the proventriculus and gizzard for solids, nor liquids, between diets, which is in accordance with the findings by van Krimpen et al. (2011). Furthermore, the estimated time of peak of OH-particles in the ceca, as estimated from the pulse-dosed Cr-mordanted OH (Table 6), as well as the area under curve (data not shown), did not differ between chickens fed fine or coarse OH. Moreover, in line with a previous broiler study (Abdollahi et al., 2019), AID of DM, nitrogen, starch, and fat were not affected by OH particle size. It should be noted that both our study and the study of Abdollahi et al. (2019) considered, high OH-inclusion levels when compared to typical commercial practices for laying hens (Ben-Mabrouk et al., 2022).

In conclusion, the results of our study indicated that in high-fiber diet with 150 g/kg oat hulls, the particle size of OH had only marginal effects on digestive processes. Coarse OH increased gizzard weight as expected, but did not affect nutrient digestibility nor digesta retention time throughout the gastrointestinal tract segments. Nevertheless, interesting variation between breeds was observed. Bovans Black hens had greater relative gizzard weight than Dekalb White hens, coinciding with greater ileal nitrogen digestibility and longer retention of solid and liquid digesta in the foregut (proventriculus and gizzard) and small intestine. Bovans Black also had higher AID of starch, but only when fed the fine OH diet. However, the total tract digestibility of NSP in Bovans Black hens was smaller, whilst having larger ceca and colon and longer MRT of liquid digesta in those segments compared with Dekalb White hens. These findings suggest that when formulating diets containing fiber-rich ingredients, regardless of their particle sizes, the variability among laying hen breeds should be taken into consideration.

## DISCLOSURES

This study was performed within the framework of the research program Innovational Research Incentives Scheme Veni with project number 15948, which is financed by the Netherlands Organisation for Scientific Research (NWO), Trouw Nutrition, and Wageningen University & Research. The authors declare that there are no conflicts of interest related to the research presented in this manuscript.
